# Calu-3 epithelial cells exhibit different immune and epithelial barrier responses from freshly isolated primary nasal epithelial cells in vitro

**DOI:** 10.1186/s13601-018-0225-8

**Published:** 2018-09-12

**Authors:** Katleen Martens, Peter W. Hellings, Brecht Steelant

**Affiliations:** 1 Department of Microbiology and Immunology, Laboratory of Clinical Immunology, KU Leuven, Herestraat 49, Box 811, 3000 Louvain, Belgium; 2 Clinical Department of Otorhinolaryngology, Head and Neck Surgery, University Hospitals Leuven, Louvain, Belgium; 3 Department of Otorhinolaryngology, Academic Medical Center, University of Amsterdam, Amsterdam, The Netherlands

**Keywords:** Primary nasal epithelial cells, Calu-3 epithelial cell line, House dust mite extract, *Staphylococcus aureus* enterotoxin B, Trans-epithelial electrical resistance

## Abstract

Epithelial cell lines are often used to evaluate the effect of exogenous/endogenous stimuli on epithelial barrier function and innate immune responses in allergic airway diseases, without clear view on differences between epithelial cell lines and primary nasal epithelial cell responses. In this observational study, we compared the response of Calu-3 and primary nasal epithelial cells to two relevant exogenous stimuli: i.e. *Staphylococcus aureus* enterotoxin B (SEB) and house dust mite (HDM). Stimulation of Calu-3 cells with SEB decreased epithelial integrity in a dose dependent manner, which was associated with a significant increase in IL-6 and IL-8 production. In contrast, no alteration in barrier integrity or IL-6 and IL-8 production was seen when primary nasal epithelial cells were stimulated with SEB. HDM extract altered the integrity of primary nasal epithelial cells, but not of Calu-3 epithelial cells. Increased IL-8 production was seen after stimulation with HDM in primary nasal epithelial cells and not in Calu-3 epithelial cells. In conclusion, immune and barrier function differ between different epithelial cell types studied. As a consequence, care must be taken when interpreting data using different epithelial cell types.

**Electronic supplementary material:**

The online version of this article (10.1186/s13601-018-0225-8) contains supplementary material, which is available to authorized users.

Airway epithelial cells serve as the first site of contact to exogenous stimuli such as dust, pollutants or microorganisms. Consequently, airway epithelial cells present a dual function, i.e. preventing the invasion of foreign particles by creating a physical barrier and defending the body by inducing an appropriate immune response. Inter-epithelial junctions like tight junctions seal off the paracellular space between the airway epithelial cells and thus protect the internal environment from the penetration of possible harmful substances [1, 2]. Previous studies revealed disturbed expression and regulation of tight junctions with impaired epithelial barrier function in asthma and allergic rhinitis [3, 4]. Consequently, studying the regulation of airway epithelial barrier function by exogenous and/or endogenous stimuli has become a major interest in better understanding the pathology of multiple airway diseases. Beside from primary bronchial or nasal epithelial cells, immortalized epithelial cell lines such as Calu-3, 16HBE or T84 cells are often used [1]. Despite the easy maintenance and unlimited amount of cells, epithelial cell lines differ in cellular responses, morphology and biochemical characteristics compared to primary nasal epithelial cells. Hence, care must be taken when interpreting data generated using cell lines as they might respond differently compared to primary nasal epithelial cells.

In this study, we report differences in response of Calu-3 epithelial cells to exogenous stimuli *Staphylococcus aureus* enterotoxin B (SEB) and house dust mite (HDM) extract compared to primary nasal epithelial cells. SEB has immunomodulatory properties in allergic airway disease [5] and can modulate barrier integrity of the intestinal epithelial cell line, T84 cells [6]. Details on methodology are provided in an Additional file 1. Therefore, we first evaluated the effect of SEB on epithelial barrier integrity in vitro. Air–liquid interface (ALI) cultures of freshly isolated primary nasal epithelial cells and Calu-3 epithelial cells were stimulated for 4 h with different concentrations of SEB (1 µg and 10 µg). Stimulation with SEB constitutively decreased trans-epithelial electrical resistance (TER) of ALI cultures of Calu-3 epithelial cells in a dose and time dependent manner (Fig. 1a). In parallel, diffusion of FITC-dextran 4 kDa (FD4) across the epithelial monolayer, as a surrogate marker for paracellular permeability, was significantly increased at time point 4 h with the highest concentration of SEB (10 µg) (Fig. 1a). On the contrary, when ALI cultures from primary nasal epithelial cells were stimulated for 4 h with SEB, no effect on TER or FD4 permeability was observed (Fig. 1b). Additionally, exposure of epithelial cells to SEB promotes the secretion of pro-inflammatory cytokines such as IL-6 and IL-8 [7]. SEB stimulation of Calu-3 epithelial cells significantly increased the release of IL-6 and IL-8, while SEB had no effect on cytokine secretion from primary nasal epithelial cells (Fig. 1c). This data demonstrates that SEB affects epithelial integrity and cytokine secretion of Calu-3 epithelial cell cultures, which is not seen in primary nasal epithelial cell cultures.

**Fig. 1:**
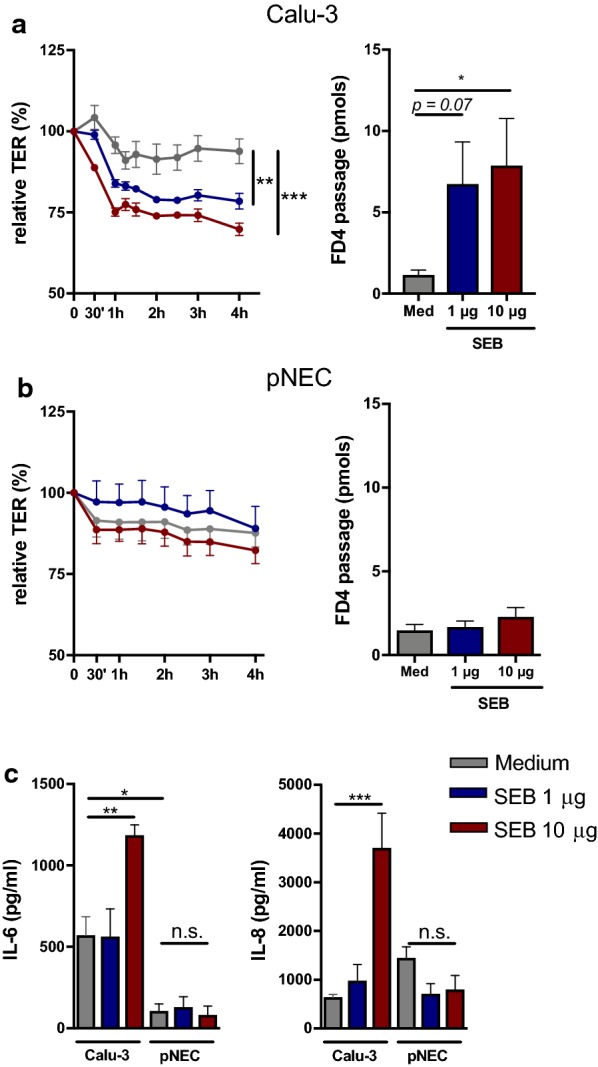
Effect of *S. aureus* enterotoxin B on epithelial cells in vitro. Primary nasal epithelial cells (n = 5) and Calu-3 epithelial cells (n = 3) at air–liquid interface were stimulated for 4 h with different concentrations of SEB. **a** Evaluation of trans-epithelial electrical resistance (TER) for 4 h of Calu-3 epithelial cell cultures. FD4 permeability of Calu-3 epithelial cells at time point 4 h. **b** Evaluation of TER for 4 h of primary nasal epithelial cells from heathy controls. FD4 permeability of primary nasal epithelial cells at time point 4 h. **c** Concentration of IL-6 and IL-8 in the supernatant of SEB stimulated Calu-3 or primary nasal epithelial cell cultures. Data presented as mean ± SEM. One-way ANOVA with post hoc test. *p < 0.05; **p < 0.01; ***p < 0.001; *ns* not significant

We next evaluated the effect of HDM on the two different epithelial cell culture systems. HDM is the most common allergen in allergic rhinitis and contains many components that act on airway epithelial cells to disrupt cell–cell contact [8]. ALI cultures of Calu-3 and primary nasal epithelial cells were stimulated with different concentrations of HDM extract (0.2, 2 and 20 µg) for 4 h. HDM did not alter TER nor FD4 permeability in ALI cultures from Calu-3 epithelial cells (Fig. 2a). However, HDM disrupted epithelial barrier integrity of ALI cultures of primary nasal epithelial cells in a dose dependent manner (Fig. 2b). In line, FD4 permeability was increased with increasing dose of HDM extract (Fig. 2b). Lastly, we evaluated the effect of HDM on cytokine production (i.e. IL-6, IL-8 and TNF-α) of epithelial cells and found that HDM did not alter IL-8 production in Calu-3 epithelial cells, while IL-8 production of primary nasal epithelial cells was elevated without reaching significance (Fig. 2c). TNF-α and IL-6 production was below detection limit for both Calu-3 and primary nasal epithelial cells (data not shown). Collectively, HDM extract impaired epithelial barrier function of primary nasal epithelial cells but not of calu-3 cells.

**Fig. 2:**
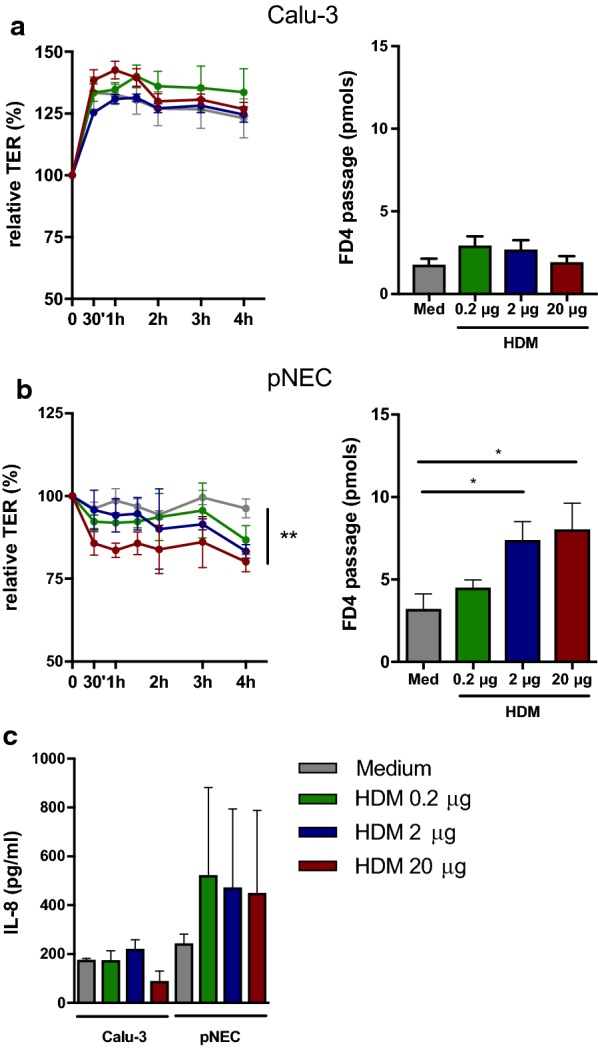
Effect of House dust mite extract on epithelial cells in vitro. Primary nasal epithelial cells (n = 5) and Calu-3 epithelial cells (n = 3) at air–liquid interface were stimulated for 4 h with different concentrations of house dust mite (HDM) extract. **a** Evaluation of trans-epithelial electrical resistance (TER) for 4 h of Calu-3 epithelial cell cultures. FD4 permeability of Calu-3 epithelial cells at time point 4 h. **b** Evaluation of TER for 4 h of primary nasal epithelial cells from heathy controls. FD4 permeability of primary nasal epithelial cells at time point 4 h. **c** Concentration of IL-8 in the supernatant of HDM stimulated Calu-3 or primary nasal epithelial cell cultures. Data presented as mean ± SEM. One-way ANOVA with post hoc test. *p < 0.05; **p < 0.01; ***p < 0.001; *ns* not significant

In the present study, we show a different response of Calu-3 epithelial cells to SEB and HDM extract compared to primary nasal epithelial cells. SEB is a superantigen with immune-modulatory and pro-inflammatory effects that can aggravate allergic airway responses [7]. In this study, stimulation of Calu-3 epithelial cells with SEB decreased barrier integrity, which was not found in primary nasal epithelial cell cultures. The decrease in barrier integrity in the Calu-3 cells was associated with an increase in IL-6 and IL-8 production. On the other hand, HDM extract impaired barrier integrity of primary nasal epithelial cells, which is in line with previous published work [9]. Surprisingly, HDM extract could not impair Calu-3 epithelial integrity, nor alter cytokine production both in Calu-3 and primary epithelial cells. We believe that these differences are associated with altered biochemical and cellular properties after immortalization of Calu-3 epithelial cells. At ALI, Calu-3 epithelial cells form a confluent, polarized monolayer with tight junction expression and a uniform mucus layer. However, we have some evidence that expression of tight junction varies in Calu-3 epithelial cells compared to primary nasal epithelial cells, which might explain the different responses to SEB and HDM. Moreover, the presence of ciliated cells and innate pattern recognition receptors on Calu-3 cells is contradictory, and thus can influence epithelial cytokine production [10]. Of note, our findings are only applicable to Calu-3 epithelial cells. The effect of exogenous stimuli on other airway epithelial cell lines has not been investigated.

In conclusion, care must be taken when studying epithelial barrier function and immune responses using epithelial cell lines as they differ in cellular responses compared to primary epithelial cells. We therefore propose, depending on the scientific research question and the availability of patient material, to use freshly isolated primary nasal epithelial cells, as disease-related processes can easily be studied and thus will provide a better understanding of the pathology of different airway diseases.

## Additional file

**Additional file 1.** The full methodology can be found in the online supplement of this manuscript.
