# Supplementary material for: Calu-3 epithelial cells exhibit different immune and epithelial barrier responses from freshly isolated primary nasal epithelial cells in vitro
Source: Clin Transl Allergy. 2018 Sep 12;8:40. doi: 10.1186/s13601-018-0225-8 (PMC6134498; doi:10.1186/s13601-018-0225-8)
Supplement: Supplementary file 1 — Additional file 1. The full methodology can be found in the online supplement of this manuscript. [file 13601_2018_225_MOESM1_ESM.docx]

# Online repository

**Title page**

Letter to the editor

**Title: Calu-3 epithelial cells exhibit different immune and epithelial barrier responses from freshly isolated primary nasal epithelial cells *in vitro***

**Authors**

Katleen Martens; MSc ^a^; Peter W Hellings, MD, PhD ^a, b, c^; Brecht Steelant, PhD

**Affiliations**

^a^ KU Leuven, Department of Microbiology and Immunology, Laboratory of Clinical Immunology, Leuven, Belgium; ^b^ University Hospitals Leuven, Clinical Department of Otorhinolaryngology, Head and Neck Surgery, Leuven, Belgium; ^c^ Department of Otorhinolaryngology, Academic Medical Center, University of Amsterdam, Amsterdam, The Netherlands

Katleen Martens [katleen.martens@kuleuven.be](mailto:katleen.martens@kuleuven.be)

Peter Hellings [peter.hellings@kuleuven.be](mailto:peter.hellings@kuleuven.be)

Brecht Steelant [brecht.steelant@kuleuven.be](mailto:brecht.steelant@kuleuven.be)

**Address for correspondence**:

Dr. Brecht Steelant

Laboratory of Clinical Immunology

KU Leuven

Herestraat 49, box 811, 3000 Leuven, Belgium

Tel.: +32 16 34 61 37

Fax: +32 16 34 60 35

E-mail: brecht.steelant@kuleuven.be

# Materials & Methods

**Isolation of primary nasal epithelial cells**

Inferior turbinates from non-allergic, non-smoking, therapy negative controls subject, were used for isolation of primary nasal epithelial cells. A highly purified epithelial cell population was obtained as reported previously (1). Briefly, tissue was washed in sterile saline and enzymatically digested in 0.1 % pronase (Protease XIV, Sigma, Belgium) solution in DMEM-F12 culture medium supplemented with 100 U/ml penicillin, 100 μg/ml streptomycin, and 2 % Ultroser G (Pall Life Sciences, Belgium). After overnight incubation at 4 °C while shaking, the protease reaction was stopped by the addition of FCS (10 %). Cells were washed in culture medium and pelleted by centrifugation for 5 min at 100 g. Cells were then resuspended in 10 ml culture medium and incubated in a plastic culture flask for 1h at 37 °C to remove the fibroblasts. The cell suspension was mixed with 2 x 10^7^ prewashed CD45- and CD15-magnetic beads (Dynabeads®, Invitrogen, Merelbeke, Belgium) and nasal epithelial cells were purified by negative selection following the manufacturer’s instructions. Diff-Quick staining was used to determine the morphology of isolated epithelial cells.

**Calu-3 epithelial cell line**

Calu-3 cells were cultured in EMEM medium (Lonza), supplemented with 10 % FCS, 1 % L-glutamine and 100 U/ml penicillin, 100 μg/ml streptomycin. The cells were plated in T75 culture flasks. Cells were split 1/3 before reaching confluence using a 0.25 % trypsin solution in EDTA (Sigma-Aldrich, St Louis, Missouri, USA). The culture medium was changed every 2 days. Calu-3 cells between passages 10-20 were used in these experiments.

**Air-liquid interface (ALI) cultures and trans-epithelial resistance (TER) measurement**

Freshly isolated primary nasal epithelial cells or Calu-3 epithelial cells were seeded on 0.4 µm 0.33 cm² polyester transwell inserts (Costar, Corning, NY, USA) at a density of 10^5^ cells/transwell. Medium was refreshed every other day. Once the cells grew to complete confluence, the apical culture medium was removed to allow further cell differentiation in ALI. At day 21 in ALI, epithelial integrity was evaluated by TER measurements using an EVOM/Endohm (WPI Inc, Sarasota, USA). Wells not building up sufficiently (TER < 200 Ω cm²) were not included in experiments. TER was measured in triplicate for each subject.

**Paracellular flux measurement**

Fluorescein isothiocyanate dextran 4 kDa (FD4) (Sigma-Aldrich, St Louis, Missouri, USA) was used to measure epithelial permeability. FD4 (2 mg/mL) was added apically to the ALI cultures at and the FITC intensity of basolateral fluid at time point 4 hours was measured using a fluorescence reader (FLUOstar Omega; BMG Labtech, Ortenberg, Germany). FD4 concentration was calculated and is expressed in pmol.

**Stimulation experiments**

Primary nasal epithelial cells were isolated from 5 different donors. Experiments were performed in triplicate. Calu-3 experiments were as well performed in triplicate and were repeated 3 times using cells between passage 10-20. ALI cultures of Calu-3 or primary nasal epithelial cell were stimulated at day 21 with 1 or 10 µg SEB (Sigma-Aldrich, St Louis, Missouri, USA) or 0.2, 2, 20 µg extract (Greer Laboratories, Lenoir, North Carolina, USA) for 4 hours. As negative control, cells were stimulated with vehicle (saline in which SEB or HDM was dissolved). For TER measurements, cells were taken out of the incubator for 5 minutes to adapt to room temperature. TER was measured at different time points and is presented as percentage change from time point 0.

**Detection of cytokines in supernatant of stimulated epithelial cells**

*Cytokines IL-6, IL-8 and TNF-α were measured in the supernatant of stimulated Calu-3 and primary nasal epithelial cells by sandwich ELISA. Plates were first coated with the capture monoclonal antibodies mouse anti-human Il-8 (554716), rat anti-human IL-6 (554546) or mouse anti-human TNF-α (551220) and incubated overnight. As secondary antibody, we used the biotinylated detection antibodies anti-IL-8 (554718, BD, Bioscience, San Diego, California), anti-IL-6 (554546, BD, Bioscience, San Diego, California) and anti-TNF-α (554511, BD, Bioscience, San Diego, California). A standard curve was made using recombinant hIL-8 (554609, BD, Bioscience, San Diego, California), recombinant hIL-6 (550071, BD, Bioscience, San Diego, California) or recombinant hTNF-α (551220, BD, Bioscience, San Diego, California).*

**Statistical analysis**

Data were analyzed using Graphpad Prism 7 (La Jolla, CA 92037 USA). Differences between groups were analyzed using a two-tailed one-way ANOVA or Kruskal-Wallis test with post hoc analysis. Two-way ANOVA was used to evaluate the effect of different stimuli in function of time. Data are presented as mean ± SEM. Values were considered significantly different when p< 0.05.

# References

1. Bobic S, van Drunen CM, Callebaut I, Hox V, Jorissen M, Fokkens WJ, et al. Dexamethasone-induced apoptosis of freshly isolated human nasal epithelial cells concomitant with abrogation of IL-8 production. Rhinology. 2010;48(4):401-7.
